# The development of endoplasmic reticulum-related gene signatures and the immune infiltration analysis of sepsis

**DOI:** 10.3389/fimmu.2023.1183769

**Published:** 2023-06-06

**Authors:** Yi Zhou, Yifang Chen, Jianbo Li, Zailin Fu, Qian Chen, Wei Zhang, Huan Luo, Minghua Xie

**Affiliations:** ^1^ Department of Pharmacy, First People’s Hospital of Linping District, Hangzhou, Zhejiang, China; ^2^ Department of Critical Care Medicine, Chongqing General Hospital, Chongqing, China

**Keywords:** sepsis, endoplasmic reticulum, machine learning, immune infiltration, diagnostic biomarkers

## Abstract

**Background:**

Sepsis is a complex condition involving multiorgan failure, resulting from the hosts’ deleterious systemic immune response to infection. It is characterized by high mortality, with limited effective detection and treatment options. Dysregulated endoplasmic reticulum (ER) stress is directly involved in the pathophysiology of immune-mediated diseases.

**Methods:**

Clinical samples were obtained from Gene Expression Omnibus datasets (i.e., GSE65682, GSE54514, and GSE95233) to perform the differential analysis in this study. A weighted gene co-expression network analysis algorithm combining multiple machine learning algorithms was used to identify the diagnostic biomarkers for sepsis. Gene Ontology (GO) analysis, Kyoto Encyclopedia of Genes and Genomes (KEGG) enrichment, and the single-sample gene set enrichment analysis algorithm were used to analyze immune infiltration characteristics in sepsis. PCR analysis and western blotting were used to demonstrate the potential role of *TXN* in sepsis.

**Results:**

Four ERRGs, namely SET, LPIN1, TXN, and CD74, have been identified as characteristic diagnostic biomarkers for sepsis. Immune infiltration has been repeatedly proved to play a vital role both in sepsis and ER. Subsequently, the immune infiltration characteristics result indicated that the development of sepsis is mediated by immune-related function, as four diagnostic biomarkers were strongly associated with the immune infiltration landscape of sepsis. The biological experiments in vitro and vivo demonstrate TXN is emerging as crucial player in maintaining ER homeostasis in sepsis.

**Conclusion:**

Our research identified novel potential biomarkers for sepsis diagnosis, which point toward a potential strategy for the diagnosis and treatment of sepsis.

## Introduction

Sepsis is a systemic inflammatory disease that remains a major global challenge owing to its high morbidity and mortality rates (1). It is defined as a highly complex infection-induced illness characterized by multiorgan failure. The annual global prevalence of sepsis is over 30 million, with the in-hospital mortality rate estimated at 20%–30% (2). Conventional wisdom has it that the activation of sepsis is associated with an overzealous inflammatory or even immune suppression response to invading pathogens (3). It is generally admitted that excessive activation of the immune response could trigger the complement system, immune dysfunction, metabolic alterations, and the cardiovascular, neurologic, and coagulation cascade, which can ultimately lead to multiple organ dysfunction, aggravating the patient’s illness, and even death (4, 5). As a major challenge in acute care medicine and surgery, no drugs that are convincingly effective have been approved specifically for the clinical treatment of sepsis as yet (6). Therefore, there is a great need to find novel and efficient approaches for the diagnosis and treatment of sepsis.

The endoplasmic reticulum (ER) is a membrane-bound organelle dedicated to the synthesis, folding, and maturation of proteins (7). It performs a vital role in aspects of eukaryotic cell physiology, such as ER calcium homeostasis, hypoxia, inflammation, apoptosis, lipid biosynthesis, and DNA damage (8). Disturbances in ER homeostasis cause the accumulation of unfolded or misfolded proteins in the ER lumen and impaired ER function, which is defined as ER stress (9). An increasing number of studies are showing that ER stress contributes to the progression of various diseases, including obesity, cancer, degenerative disorders, and immune-mediated diseases (10, 11). Dysregulated ER stress is related to inflammation (12). It is widely established that sepsis is a systemic inflammatory response, and the core mechanism of initiation and progression of sepsis is inflammation (13). Consequently, a better understanding of the role of the ER in the progression of sepsis is essential so as to identify novel diagnostic markers and therapeutic targets for sepsis.

The detection of biomarkers promises to offer improved opportunities for disease diagnosis, prognosis, and prevention (14). Therefore, focusing on the role of ER-related genes (ERRGs) in sepsis is becoming crucial in fully understanding the diagnosis, evaluation, and treatment of sepsis. In the present study, we aimed to investigate the association between ERRGs and sepsis based on the GeneCards^®^ database, Gene Ontology (GO) analysis, and Kyoto Encyclopedia of Genes and Genomes (KEGG) enrichment. Four intersection genes were identified as diagnostic biomarkers for sepsis. Furthermore, the relationship between the four diagnostic biomarkers and the immune infiltration characteristic of sepsis was comprehensively explored. The present study is expected to provide novel ideas and biomarkers for the clinical diagnosis and treatment of sepsis.

## Materials and methods

### Gene Expression Omnibus dataset collection

Three publicly accessible datasets of healthy samples and sepsis samples were obtained from the Gene Expression Omnibus database. A total of 42 healthy samples and 760 sepsis samples were obtained from GSE65682 for the training cohort. Then, samples retrieved from GSE54514 and GSE95233 were used for the test cohort, in which 58 healthy samples and 229 sepsis samples were included. Under the R language, the “sva” package was adopted to remove the batch effect of the expression matrix and normalize the arrays of GSE65682, GSE54514, and GSE95233. The ERRGs were acquired from the GeneCards database (https://www.genecards.org/) (**Supplementary Table 1**).

### Exploration of differentially expressed genes and functional enrichment analysis

For the healthy samples and sepsis samples in the training cohort, the levels of expression of differentially expressed genes (DEGs) were calculated, with the selection cutoff value set at a fold change ≥ 2 and a *p*-value< 0.05. The “clusterProfiler” package was adopted to explore the GO and KEGG enrichment of DEGs between the healthy and the sepsis samples.

### Weighted gene co-expression network analysis and crucial endoplasmic reticulum-related gene identification

We developed a weighted gene co-expression network analysis (WGCNA) algorithm to explore the key gene module associated with clinical traits for healthy and sepsis patients. First, the missing expression data were excluded, and abnormal genes and samples were removed from the data. With the filter threshold of data expression set at 0.5, the samples were clustered in a clustering tree and the outliers were deleted. The scale-free topology model fit was set at an *R* ^2^ value > 0.85 and the range of soft thresholding powers was set at 1–30. The scale-free network was constructed in accordance with the optimal soft thresholding power. Second, the adjacency was turned into topological overlap, and the module was identified using the dynamic tree cut algorithm. Finally, based on the calculation of Pearson correlation coefficients, the relationship between the module and clinical features was acquired and the most characteristic gene module was chosen for the final investigation. The “Venn” package was chosen to exhibit the overlapping genes from the WCGNA, DEGs, and ERRGs. The protein–protein interaction (PPI) network of the overlapping genes was explored using the STRING database (https://cn.string-db.org/).

### The generation of features variates using a machine learning algorithm

Multiple machine learning languages were adopted to explore the feature variates of the key ERRGs for sepsis. The “glmnet” package was used to run the least absolute shrinkage and selection operator (LASSO) algorithm. The “randomForest” package was used to calculate the importance of variates; the cutoff value for selecting characteristic variates was > 5. Support vector machine recursive feature elimination (SVM-RFE) was performed to explore the feature variates *via* the “e1071”, “caret”, and “kernlab” packages. Overlapping genes from LASSO, the randomForest (RF) algorithm, and SVM-RFE were detected *via* a Venn diagram and included in the next investigation.

### Consensus clustering analysis and immune infiltration characteristics evaluation

Based on the expression profile of diagnostic biomarkers, an unsupervised consensus clustering analysis was developed to explore the molecular subgroups of sepsis using the “ConsensusClusterPlus” package. With the sample distribution *k* set at 2–9, the optimal classification pattern was calculated. On the basis of an Estimation of STromal and Immune cells in MAlignant Tumours using Expression (ESTIMATE) data assessment, the immune score of sepsis subgroups was obtained. The gene markers and gene expression data for 23 immune cells were investigated. The “limma” package was used to calculate the DEGs of sepsis subgroups, with the cutoff value set at a fold change > 1, and at a *p*-value< 0.05. The “ggplot2” package was utilized to explore the distribution pattern of sepsis subgroups.

### Nomogram development and diagnostic ability evaluation

The “rms” package was used to develop the nomogram model for evaluating the diagnostic ability of *SET*, *LPIN1*, *TXN*, and *CD74* for sepsis. The nomogram score was obtained as:


(1) Score = expression of SET,LPIN1,TXN, and CD74 × coefficients

The diagnostic ability of *SET*, *LPIN1*, *TXN*, and *CD74* and the nomogram score for sepsis were evaluated using a receiver operating characteristic (ROC) curve.

### Western blotting

RIPA buffer [25 mm Tris, at pH 7.6, 150 mM NaCl, 1% NP-40, 1% sodium deoxycholate, and 0.1% sodium dodecyl sulfate (SDS)] was used to extract the total protein mass from cells. The proteins were separated by 12% sodium dodecyl sulfate-polyacrylamide gel electrophoresis and transferred to polyvinylidene fluoride membranes (0.45 mm; Millipore, Germany). After being incubated with antibodies, the membrane was visualized using an Odyssey^®^ CLx imager (LI-COR, USA). CHOP, GRP78, and GRP94 were purchased from Abcam (USA), and β-actin was purchased from Cell Signalling Technology, Inc. (USA). The intensity of the bands was analyzed by using Quantity One software (version 4.62; Bio-Rad Life Science, California, United States).

### qRT-PCR analysis

RNA was extracted from rat whole-blood samples by using TRIzol™ Reagent (Cat# 15596-026, Invitrogen, America) according to the manufacturer’s instructions. cDNA was synthesized using a PrimeScriptTM RT reagent kit (Cat#RR047A, Takara, Japan). For quantitative real-time PCR (qRT-PCR) analysis, triplicate PCR reactions were carried out for each sample in an Mx3000P real-time thermal cycler (Stratagene, America). PCR cycling was initiated with a 2-minute heating step at 94°C and then a run-through of 22–31 cycles (95°C for 10 s, 60°C for 20 s, and 72°C for 30 s) to monitor the linearity of the amplification. The sample was then kept at 72°C for 10 min, and the reaction stopped by decreasing the temperature to 4°C. The β-actin gene was used as an internal control. The quantification of relative mRNA levels was determined as described by Xue et al. (15). The primer sequences used for PCR amplification are shown in **Table 1**.

**Table 1:** Sequences of primers.

Gene	Forward primer (5′–3′)	Reverse primer (5′–3′)
*SET*	AAACCAAGACCACCTCCTGC	TTCTCCCTTCTTCGGCAAGC
*LPIN1*	GAGGAAAACCTCTCCCTGGC	CCCCACAGCCAAAGCATTTC
*TXN*	GATGTGGATGACTGTCAGGATG	TTCACCCACCTTTTGTCCCTT
*CD74*	TGGAGCAAAAGCCCACTGAC	CAGTAGCCGATGCTCCCATAG

### Statistical analysis

All statistical analyses were performed in R (version 4.1.1), GraphPad Prism (version 8.0.1), and SPSS 18.0 (SPSS Inc., Chicago, IL, USA). Student’s *t*-tests and Wilcoxon rank-sum tests were used to detect significant differences between the two groups. One-way ANOVA was used between multiple groups. A *p*-value*<* 0.05 was considered to be statistically significant. All data are presented as the mean ± standard deviation (SD).

## Results

### Analysis of differentially expressed genes and functional enrichment evaluation

**Figure 1:**
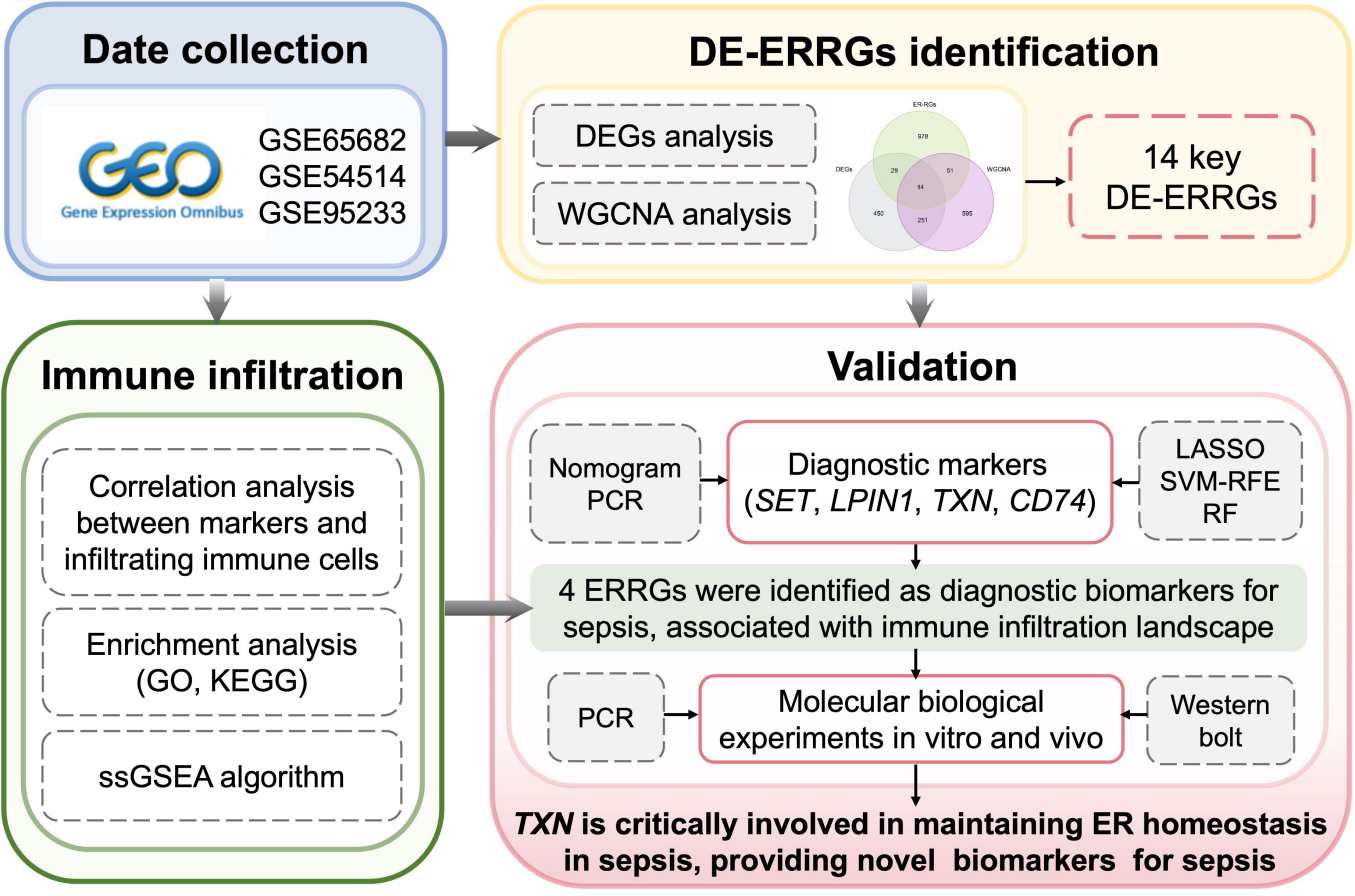
Study flow.

**Figure 2:**
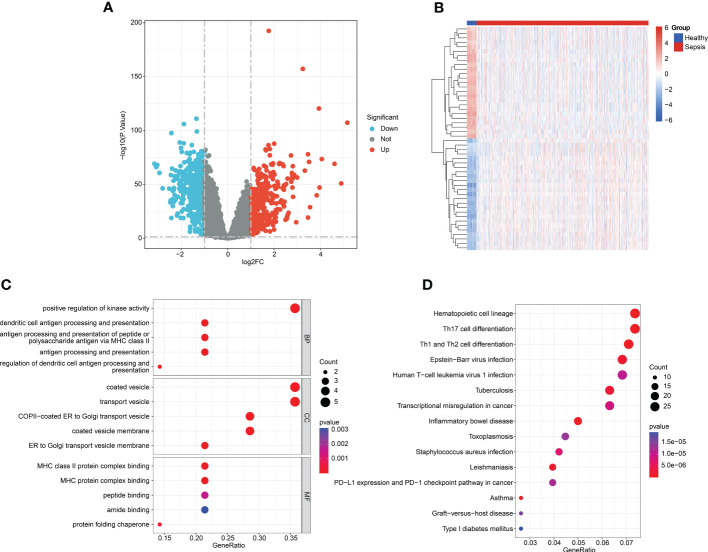
Analysis of the DEGs and molecular function enrichment. **(A)** Volcano analysis of the DEGs between healthy and sepsis groups. **(B)** Heatmap illustrating the top 25 up- and downregulated DEGs in the healthy and sepsis groups. **(C)** GO enrichment analysis of the DEGs. **(D)** KEGG enrichment analysis of the DEGs.

A total of 42 healthy samples and 760 sepsis samples were obtained from the GSE65682 dataset to perform the differential analysis in this study (**Figure 1**). With the screening criteria set at a fold change > 2 and a *p*-value< 0.05, the DEGs between the healthy and sepsis groups were acquired (**Figure 2A**). The heatmap exhibits the top 25 up- and downregulated DEGs in the healthy and sepsis groups (**Figure 2B**). To further explore the molecular function of DEGs in the development of sepsis, we conducted GO and KEGG enrichment analyses. GO enrichment analysis indicated that the DEGs were involved in the positive regulation of kinase activity, dendritic cell antigen processing and presentation, vesicle coating, vesicle transport, and MHC class II protein complex binding (**Figure 2C**). The KEGG enrichment analysis revealed that the DEGs were linked with the hematopoietic cell lineage, Th17 cell differentiation, and Th1 and Th2 cell differentiation (**Figure 2D**).

### The construction of the WGCNA algorithm to identify the crucial gene module

**Figure 3:**
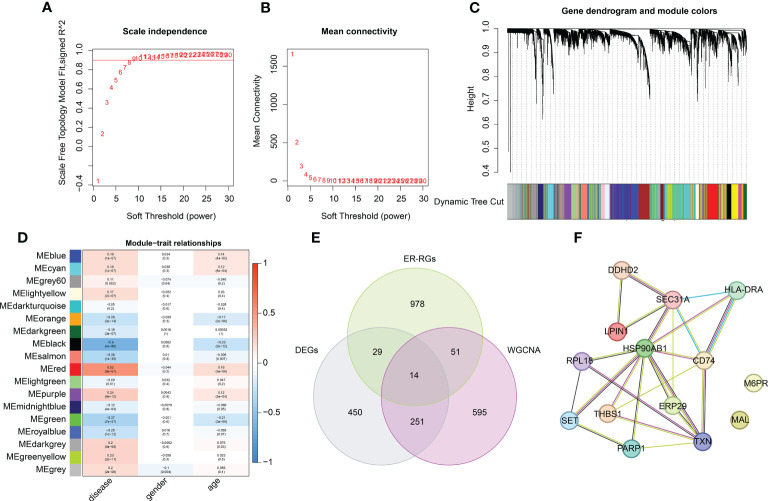
WGCNA construction of the transcriptome matrix and key DE-ERRGs selection. **(A, B)** Scale independence and mean connectivity. The soft threshold (power) β = 9. **(C)** Gene dendrogram and module color. **(D)** Association between different gene modules and clinical features. **(E)** Venn plot showing the key DE-ERRGs determined by WGCNA and differential expression analysis. **(F)** PPI network exhibiting the relationship between the 14 key DE-ERRGs.

Based on the clinical features of healthy and sepsis samples from GSE65682, we developed a WGCNA algorithm to identify the crucial gene module associated with the clinicopathological characteristics of sepsis. First, we selected the soft threshold (β = 9) to develop the scale-free network, with the scale-free topology model fit set at an *R* ^2^ value > 0.85 (**Figures 3A, B**). As shown in **Figure 3C**, a total of 18 modules were obtained by the dynamic tree cut algorithm. The heatmap illustrates a clear relationship between the 18 gene modules and clinical traits. We observed that the black module was negatively associated with disease (*r* = −0.60, *p* = 4e^–80^) and age (*r* = −0.25, *p* = 2e^–12^); the red module was positively correlated with disease (*r* = 0.52, *p* = 8e^–57^) and age *(r* = 0.16, *p *=* *5e^–6^); and the salmon module was negatively associated with disease (*r* = −0.36, *p *=* *1e^–25^) and age (*r* = −0.095, *p* = 0.007) (**Figure 3D**). Combining the correlation analysis of gene modules and clinical traits, we selected the black gene modules for subsequent analysis. As displayed in a Venn diagram, 14 intersection genes were identified as characteristic Differential Expression-Endoplasmic Reticulum-related Genes (DE-ERRGs) for sepsis (**Figure 3E**). The PPI network was used to explore the association of characteristic variates, and the result showed a clear correlation between the 14 Differential Expression-Endoplasmic Reticulum-related Genes (**Figure 3F**).

### Generation of characteristic diagnostic biomarkers *via* a machine learning algorithm

**Figure 4:**
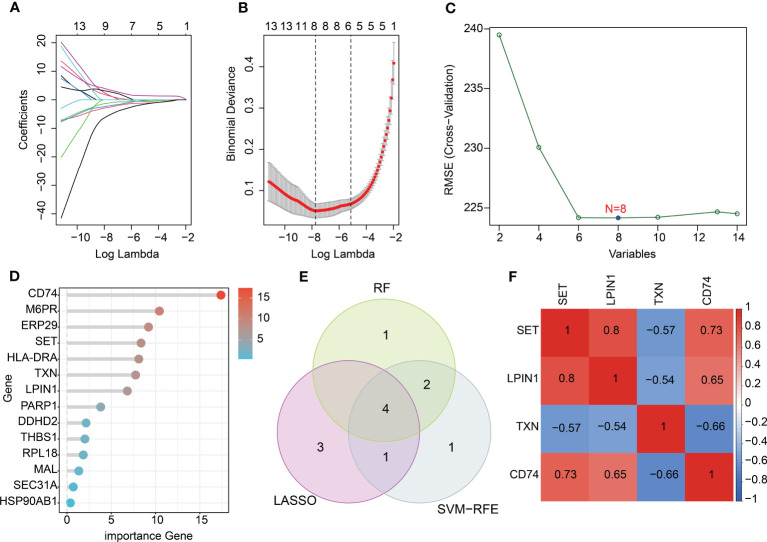
Characteristic diagnostic biomarker exploration using machine learning algorithms. **(A, B)** Identification of feature variates using LASSO analysis. **(C)** SVM-RFE analysis of 14 key DE-ERRGs. **(D)** Variate importance of DE-ERRGs determined via the RF algorithm. **(E)** Venn diagram showing the intersection genes of three machine learning algorithms. **(F)** Correlation heatmap of diagnostic biomarkers.

To further explore the crucial diagnostic biomarkers for sepsis, we developed multiple machine-learning algorithms based on 14 key DE-ERRGs. As exhibited in **Figures 4A, B**, the LASSO analysis generated the coefficients and lambda of 14 DE-ERRGs, and eight characteristic variates were identified at the minimum lambda value. In addition, the SVM-RFE algorithm selected eight variates for further investigation (**Figure 4C**). The importance of the 14 DE-ERRGs was explored *via* the RF algorithm, and seven crucial genes were screened (**Figure 4D**). Based on the three machine learning algorithms, four intersection characteristic genes were obtained as diagnostic biomarkers, including *SET*, *LPIN1*, *TXN*, and *CD74* (**Figure 4E**). The association analysis illustrated great relatedness between *SET*, *LPIN1*, *TXN*, and *CD74* (**Figure 4F**). A clear positive association was observed between *SET*, *LPIN1* (*r* = 0.8), and CD74 (*r* = 0.73), and a negative association was detected between *SET* and *TNX* (*r* = –0.57). *LPIN1* was negatively linked with *TXN* (*r* = –0.54) and positively associated with *CD74* (*r* = 0.65). *TNX* was negatively associated with *CD74* (r = –0.66).

### The exploration and verification of diagnostic effectiveness

**Figure 5:**
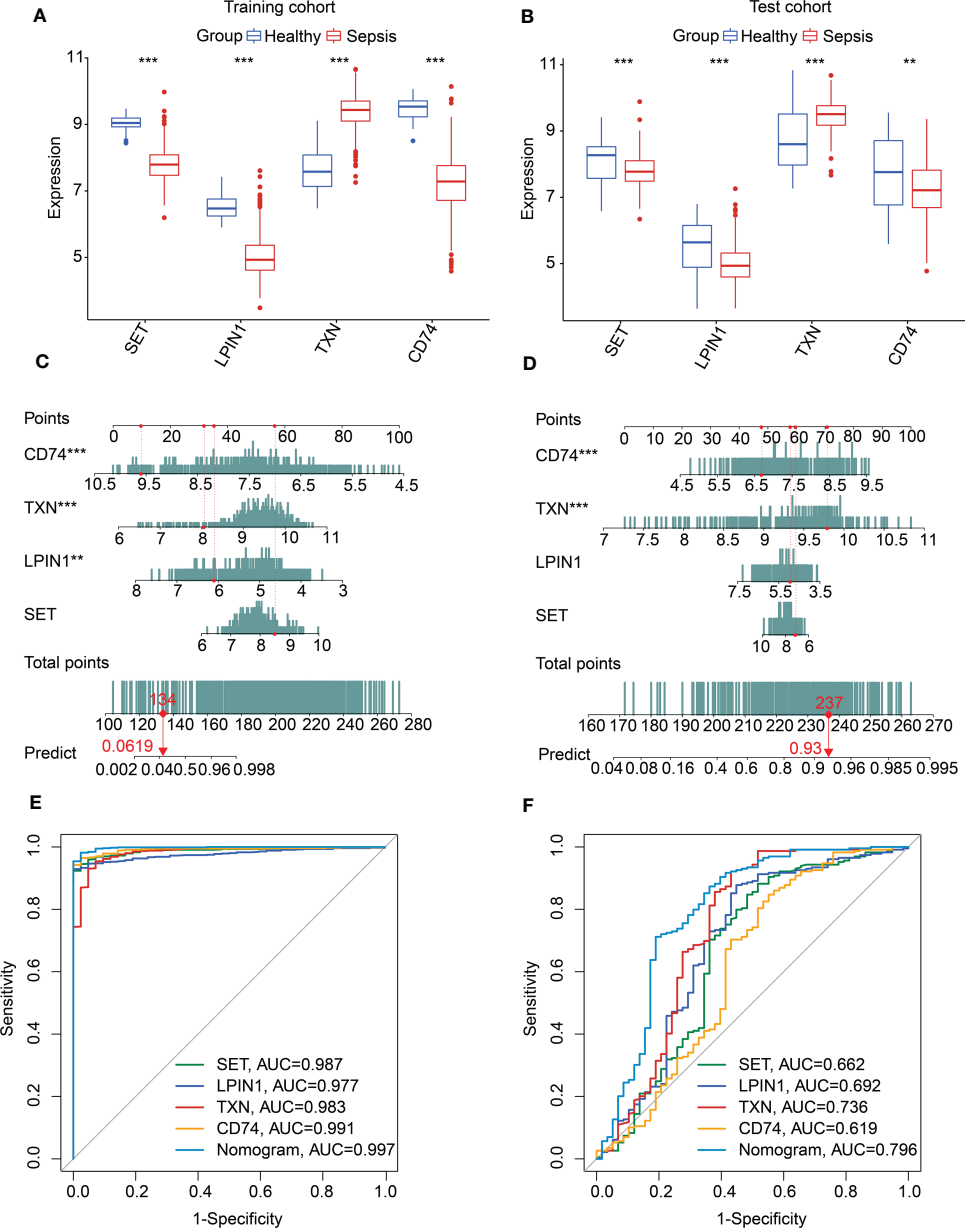
Nomogram construction and diagnostic effectiveness evaluation. Expression profile of SET, LPIN1, TXN, and CD74 in **(A)** the training cohort and **(B)** the test cohort. **(C, D)** Nomogram developed based on diagnostic biomarker expression profiles in the training and test cohorts. **(E, F)** Diagnostic effectiveness exploration of diagnostic biomarkers and nomogram.

We used the training cohort and the test cohort to explore the independence and diagnostic value of *SET*, *LPIN1*, *TXN*, and *CD74* for sepsis. In the training and test cohorts, we observed that the expression profiles of *SET*, *LPIN1*, and *CD74* were significantly higher in the healthy group, whereas the expression of *TXN* was much higher in the sepsis group (**Figures 5A, B**). In addition, a nomogram model was developed to explore the diagnostic effectiveness of the expression profiles of *SET*, *LPIN1*, *TXN*, and *CD74* for sepsis in both cohorts (**Figures 5C, D**). The ROC analysis in the training cohort suggested that the areas under the curve (AUCs) for *SET*, *LPIN1*, *TXN*, *CD74*, and the nomogram model were 0.987, 0.977, 0.983, 0.991, and 0.997, respectively (**Figure 5E**). In the test cohort, the AUCs for *SET*, *LPIN1*, *TXN*, *CD74*, and the nomogram model were 0.662, 0.692, 0.736, 0.619, and 0.796, respectively (**Figure 5F**). Taken together, these results illustrated a rewarding diagnostic value of four feature biomarkers. Moreover, the nomogram constructed based on the four diagnostic biomarkers could precisely estimate their diagnostic effectiveness for sepsis.

### The generation of molecular subtypes based on the diagnostic biomarkers

**Figure 6:**
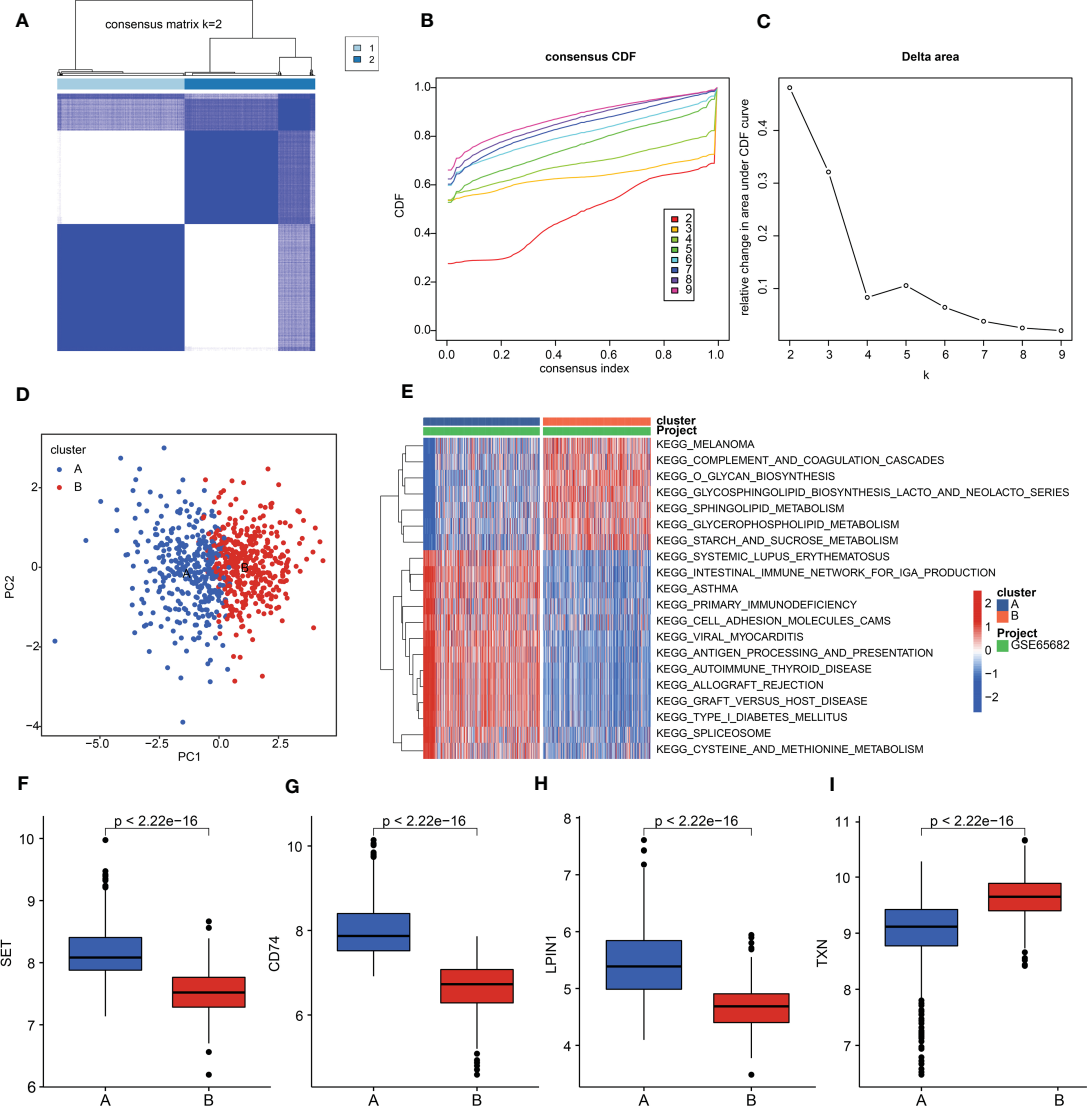
Molecular subgroups of sepsis. **(A)** Consensus matrix for k = 2. **(B)** Consensus cumulative distribution function (CDF). **(C)** Delta area. **(D)** PCA plot of clusters A and B. **(E)** GSVA estimation of KEGG terms in sepsis. **(F–I)** Expression levels of SET, LPIN1, TXN, and CD74.

To further explore the molecular subtypes of sepsis, we conducted an unsupervised consensus clustering analysis. Based on the expression profile of *SET*, *LPIN1*, *TXN*, and *CD74*, two molecular subgroups of sepsis were obtained with *K* = 2, with 375 samples in cluster A and 385 samples in cluster B (**Figures 6A-C**). A principal component analysis (PCA) diagram displayed a clear intergroup distribution pattern of sepsis in clusters A and B (**Figure 6D**). A gene set variation analysis (GSVA) was performed to explain the potential molecular mechanisms of sepsis between the subgroups. The GSVA result exhibited that several metabolism-related signaling pathways were upregulated in cluster B, such as glycerophospholipid metabolism, sphingolipid metabolism, and starch and sucrose metabolism. Notably, immune pathways were greatly downregulated in sepsis in cluster B, involving primary immunodeficiency, cell adhesion molecules, and antigen processing and presentation (**Figure 6E**). Expression analysis of four diagnostic biomarkers suggested that the sepsis samples in cluster A had higher expression of *SET*, *LPIN1*, and *CD74*; however, the expression of *TXN* was remarkably higher in cluster B (**Figure 6F–I**).

### Characteristics of immune infiltration in different molecular subgroups

**Figure 7:**
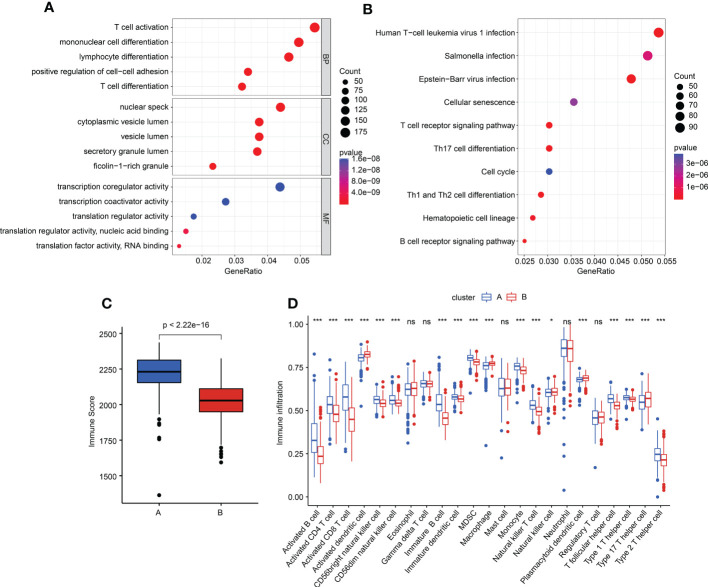
Generation of DEGs and tumor microenvironment (TME) characteristics in subgroups. **(A, B)** GO and KEGG enrichment evaluation of DEGs in different subgroups. **(C)** Immune score estimation in sepsis. **(D)** Immune infiltration feature of 23 immune cells in the sepsis subgroups.

We performed a difference analysis to explore the potential functional regulation of sepsis in cluster A and cluster B. Under the threshold criteria of a fold change > 1 and with a *p*-value< 0.05, we obtained 1,968 DEGs (1,051 upregulated and 917 downregulated) between the two subgroups *via* the “limma” package. The GO enrichment estimation result showed that T-cell activation, mononuclear cell differentiation, lymphocyte differentiation, nuclear specks, and transcription coregulator activity may mediate the function of DEGs in sepsis (**Figure 7A**). The KEGG result suggested that the DEGs were greatly enriched in human T-cell leukemia virus 1 infection, *Salmonella* infection, and Epstein–Barr virus infection (**Figure 7B**). These results implied that immune function may play a crucial role in the development of sepsis. Based on the enrichment evaluations, we further explored the immune score of sepsis in the subgroups. The result suggested that the sepsis samples in cluster B had a lower immune score than those in cluster A (**Figure 7C**). In addition, the single-sample gene set enrichment analysis (ssGSEA) algorithm was utilized to estimate the immune infiltration characteristic of sepsis in clusters A and B. The result displayed a remarkable difference in 23 kinds of immune cells between the two sepsis subgroups, such as activated B cells, CD4+ T cells, immature dendritic cells, and plasmacytoid dendritic cells (**Figure 7D**). Overall, these findings illustrated that immune function may mediate the development of sepsis.

### The association of diagnostic biomarker and Immune infiltration characteristic

**Figure 8:**
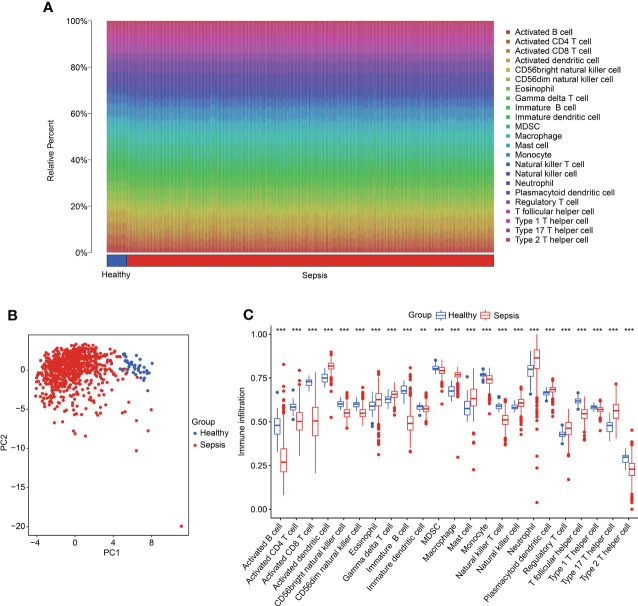
Evaluation of immune infiltration landscape in healthy and sepsis groups. **(A)** The percentage of 23 immune cells in the healthy and sepsis groups, as calculated by ssGSEA. **(B)** PCA plot based on the immune cell fractions. **(C)** The fractions of cells associated with immune infiltration in healthy and sepsis groups.

We further investigated the immune infiltration landscape in the healthy and sepsis groups. Based on a ssGSEA algorithm, the proportions of 23 immune cells were calculated in samples in the healthy and sepsis groups (**Figure 8A**). The PCA diagram revealed a clear separation between the healthy and sepsis groups based on the immune infiltration score (**Figure 8B**). The ssGSEA estimation displayed a great difference in the proportions of the 23 immune cells in samples in the healthy and sepsis groups (**Figure 8C**).

**Figure 9:**
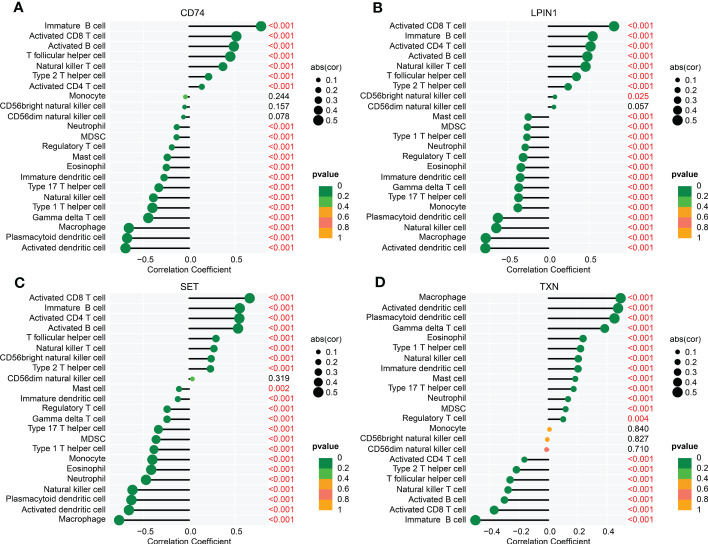
Correlation analysis of four biomarkers and immune infiltration features. The lollipop plots show the relationship between immune infiltration characteristics and CD74 **(A)**, LPIN1 **(B)**, SET **(C)**, and TXN **(D)**.

Meanwhile, we further explored the relationship between four diagnostic biomarkers and immune infiltration characteristics. As illustrated in **Figure 9**, the correlation results implied that *CD74* was linked with the presence of immature B cells, CD8+ T cells, activated B cells, and T follicular helper cells, but negatively linked with the presence of macrophages, plasmacytoid dendritic cells, and activated dendritic cells. *LPIN1* was positively associated with CD8+ T cells, immature B cells, CD4+ T cells, activated B cells, and natural killer T cells, but negatively correlated with activated dendritic cells, macrophages, natural killer cells, and plasmacytoid dendritic cells. *SET* was positively correlated with activated B cells, CD4+ T cells, immature B cells, and CD8+ T cells, but negatively correlated with macrophages, activated dendritic cells, plasmacytoid dendritic cells, and natural killer cells. *TXN* was positively associated with plasmacytoid dendritic cells, activated dendritic cells, and macrophages, but negatively associated with immature B cells. These results demonstrated a remarkable difference in healthy and sepsis groups in immune infiltration, and that this result is greatly associated with the upregulation of *CD74*, *LPIN1*, *SET*, and *TXN*.

### The inhibition of *TXN* can reduce endoplasmic reticulum stress in cardiomyocytes after sepsis

**Figure 10:**
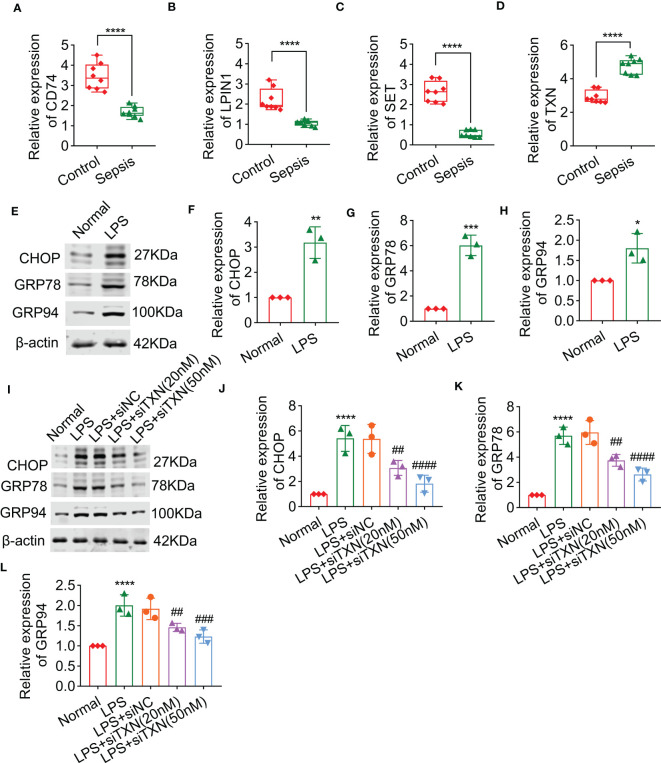
The effect of TXN on endoplasmic reticulum stress of cardiomyocytes after sepsis, including the levels of CD74 **(A)**, LPIN1 **(B)**, SET **(C)**, and TXN **(D)**, as detected by qPCR in control and sepsis rats (n = 6). **(E–H)** The expressions of CHOP, GRP78, and GRP94 after sepsis (n = 3). **(I–L)** The expressions of CHOP, GRP78, and GRP94 after being treated with siTXN (n = 3). Results are expressed as mean ± SD. Each analysis in vitro was performed in triplicate. Significance level: *p< 0.05, **p< 0.01, ***p< 0.001, and ****p< 0.0001 versus control; #p< 0.05, ##p< 0.01, ###p< 0.001, and ####p< 0.0001 versus sepsis.

Through PCR analysis, it was found that levels of *CD74*, *LPIN1*, and *SET* were significantly decreased after sepsis in rats, whereas the level of *TXN* was significantly increased after sepsis in rats, which was consistent with the results of bioinformatics (**Figures 10A-D**). To clarify the role of these key genes in the regulation of endoplasmic reticulum stress. We first used the biomarkers of ER stress (i.e., CHOP, GRP78, and GRP94) that were previously reported to observe whether or not lipopolysaccharide (LPS) stimulation can cause endoplasmic reticulum stress. The results showed that after being treated with LPS, the biomarkers of ER stress were significantly increased (**Figures 10E-H**). Because *TXN* was significantly upregulated in sepsis and had a good AUC in the training and validation sets, we also observed the effect of interfering *TXN* on ER stress after LPS stimulation. The results showed that after being treated with siTXN (20 nM or 50 nM), the expressions of CHOP, GRP78, and GRP94 were significantly decreased, and the effect of 50 nM was better (**Figures 10I-L**).

## Discussion

Sepsis has been recognized as a life-threatening illness, characterized by multiorgan dysfunction, which results in high morbidity and mortality, with limited pharmacologic therapy (13). An immunosuppressive status is the main cause of increased mortality in patients with advanced sepsis (16). Research suggests that timely diagnosis and treatment could effectively reduce the mortality of sepsis patients (17). In the current study, 14 intersection genes were identified as characteristic DE-ERRGs of sepsis. We then identified four ERRGs as diagnostic biomarkers for sepsis, through multiple machine learning algorithms, including *SET*, *LPIN1*, *TXN*, and *CD74*. The diagnostic effectiveness of these four biomarkers for sepsis was further proved by the construction of a nomogram. Notably, our investigation also found that immune-related function played a vital role in mediating the development of sepsis, while at the same time being greatly associated with four diagnostic biomarkers. Taken together, our research suggests novel potential biomarkers for sepsis diagnosis, which offers new options for the diagnosis and treatment of sepsis.

As a major site for the synthesis of membrane and secretory proteins, the ER is critical for the maintenance of intracellular protein function (18). The disruption to ER homeostasis could lead to ER stress, which has been implicated in a wide range of pathologies, including inflammatory diseases (19). Previous studies have pointed out that ER stress has the potential to result in proinflammatory signaling (20). In this study, we focused on the relationship between ERRGs and sepsis. The association analysis illustrated notable relationships between four ERRGs (including *SET*, *LPIN1*, *TXN*, and *CD74*) and sepsis. The expression profiles of *SET*, *LPIN1*, and *CD74* were significantly lower in the sepsis patients, whereas the expression of *TXN* was much higher. *SET* is a multifunctional histone chaperone localized in the nucleus predominantly, regulating chromatin condensation and transcription, and involved in many cellular processes (21). Fan et al. have proposed that, as a component complex in the ER, *SET* mediates caspase-independent nuclease activity triggered by granzyme A (22). The silencing or inhibition of *SET* impaired cell growth in a variety of human diseases (23). *LPIN1* is the major culprit in metabolic myopathies, mitochondrial diseases, abnormal lipid metabolism, and inflammation (24). It could encode lipin-1, dephosphorylating phosphatidic acid to form diacylglycerol in the ER (24). It was reported that *CD74* is an attractive candidate biomarker for immunotherapy (25). Various research had indicated that *CD74* was expressed abnormally in inflammatory disorders, whereas its role in sepsis was still unknown (25). Thioredoxin (TXN) is a ubiquitous oxidoreductase regenerated from TXNRD2. Previous studies have proposed that the knockdown of *TXN* could inhibit cell proliferation (26). *TXN* is considered critical for the inflammatory condition, which is being investigated for the treatment of many diseases (26). Previous studies have suggested that *TXN* is involved in the immune response and oxidation–reduction processes. It participated in the defense against oxidative stress by controlling cellular free radicals and reactive oxygen species (27, 28). The aforementioned studies implied that the four ERRGs were expressed abnormally in inflammation-related diseases especially, indicating the potential diagnostic value of four feature biomarkers in sepsis. Subsequently, the nomogram model we constructed confirmed the diagnostic effectiveness of the four ERRGs for sepsis.

Immune response has been repeatedly shown to play a vital role both in sepsis and ER stress (29, 30). Sepsis perturbs immune homeostasis in humans (31). Based on the expression profile of the four ERRGs, we divided the patients with sepsis into two molecular subgroups. After the analysis of immune infiltration characteristics in the two molecular subgroups, the immune-related function was proven to play an essential role in the development of sepsis, which is in concordance with previous literature (32, 33). Notably, a remarkable difference was found in 23 kinds of immune cells between the two sepsis subgroups, especially activated B cells, CD4+ T cells, immature dendritic cells, and plasmacytoid dendritic cells. Previous research has shown that T/B cells displayed immune dysfunction, which was linked to increased morbidity and mortality in sepsis (34). Dendritic cell apoptosis was a central pathogenic event during sepsis-induced immunosuppression (35). Meanwhile, the correlation results further verified the association between the four ERRGs and immune cells. Overall, the findings indicated the immune status of patients with sepsis strongly suggested that the four diagnostic biomarkers were strongly associated with the immune infiltration landscape.

Another supportive fact for our study was that the mRNA expression levels of *CD74*, *LPIN1*, and *SET* were downregulated in rats with sepsis, whereas *TXN* was upregulated. Previous studies have shown that CHOP, GRP78, and GRP94 were considered a hallmark of ER stress (36, 37). *CHOP* and *GRP78* are the critical transcriptional genes of molecular chaperones in the ER, which could be activated by the ER stress response (38). The expression of CHOP could eventually be triggered by ER stress, while GRP78 and GRP94 are upregulated (39). In the present study, we found that the ER stress could be triggered by LPS-induced sepsis. The protein abundance and mRNA levels of CHOP, GRP78, and GRP94 were significantly upregulated in LPS-induced sepsis, consistent with reports in the previous literature. Interestingly, the aforementioned phenotypes of ER stress in sepsis may be rescued by *TXN* knockdown. Given the above findings, it seems likely that *TXN*, a unique ERRG in sepsis, is emerging as an important player in sepsis diagnosis.

Taken together, four ERRGs were identified as diagnostic biomarkers for sepsis in the present study, including *SET*, *LPIN1*, *TXN*, and *CD74*. The analysis of immune infiltration further proved the immune mechanism in sepsis pathogenesis, providing strong support for four biomarkers for sepsis diagnosis. The results of molecular biological experiments *in vitro* and *in vivo* further support the fact that TXN is a key player in maintaining ER homeostasis in sepsis. Collectively, our research provides novel potential biomarkers and offers new options for the diagnosis and treatment of sepsis.

## Data availability statement

The original contributions presented in the study are included in the article/**Supplementary Material**. Further inquiries can be directed to the corresponding authors.

## Ethics statement

The animal study was reviewed and approved by First People’s Hospital of Linping District, Hangzhou.

## Author contributions

YZ and MX created the study concept and design. JL and QC contributed to data collection and data analysis. YZ and ZF conceived the original ideas and composed this manuscript. YZ, YC, and WZ contributed to the table and figures in this manuscript. YC and HL supervised the study and the revision of the article. All authors contributed to the article and approved the submitted version.
